# The Interplay Between Cervicovaginal Microbiota Diversity, *Lactobacillus* Profiles and Human Papillomavirus in Cervical Cancer: A Systematic Review

**DOI:** 10.3390/healthcare13060599

**Published:** 2025-03-10

**Authors:** Giosuè Giordano Incognito, Carlo Ronsini, Vittorio Palmara, Paola Romeo, Giuseppe Vizzielli, Stefano Restaino, Marco La Verde, Orazio De Tommasi, Marco Palumbo, Stefano Cianci

**Affiliations:** 1 Department of General Surgery and Medical Surgical Specialties, University of Catania, 95123 Catania, Italy; mpalumbo@unict.it; 2 Department of Woman, Child and General and Specialized Surgery, University of Campania “Luigi Vanvitelli”, 80138 Naples, Italy; carlo.ronsini@unicampania.it (C.R.); marco.laverde88@gmail.com (M.L.V.); 3 Unit of Gynecology and Obstetrics, Policlinico “G. Martino”, Department of Human Pathology of Adult and Childhood “G. Barresi”, University of Messina, 98122 Messina, Italy; vittorio.palmara@unime.it (V.P.); paolaromeo135@gmail.com (P.R.); stefanoc85@hotmail.it (S.C.); 4 Department of Maternal and Child Health, Obstetrics and Gynecology Clinic, University Hospital of Udine, 33100 Udine, Italy; giuseppevizzielli@yahoo.it (G.V.); restaino.stefano@gmail.com (S.R.); 5 Division of Gynecologic Oncology, Catholic University of the Sacred Heart, 00168 Rome, Italy; 6 PhD School in Biomedical Sciences, Gender Medicine, Child and Women Health, University of Sassari, 07100 Sassari, Italy; 7 Department of Women and Children’s Health, Clinic of Gynecology and Obstetrics, University of Padua, 35100 Padua, Italy; odetommas@gmail.com

**Keywords:** cervical cancer, microbiota, human papillomavirus, cancerogenesis, gynecological cancer

## Abstract

**Background and Objectives:** Interest in defining the characteristics of the cervicovaginal microbiota (CVM) in invasive cervical cancer (ICC) is growing, particularly concerning *Lactobacillus* species, as evidence suggests that these may differ in affected women and potentially interact with Human Papillomavirus (HPV) infection. Understanding these features could have important implications for disease management. Thus, this study aims to systematically review the main characteristics of available literature exploring the relationship between CVM diversity, *Lactobacillus* profiles, and HPV in ICC; **Methods:** A comprehensive bibliographic search was conducted across databases, including Medline, Embase, Scopus, the Cochrane Database of Systematic Reviews, and ClinicalTrials.gov, in accordance with the to the PRISMA guidelines. The review included studies that met the following inclusion criteria: studies comparing CVM in women with ICC to controls, focusing on Community State Types (CSTs), *Lactobacillus* profiles, and microbial diversity. Exclusion criteria included commentaries, letters, reviews, and studies without control groups. Variables were analyzed using the Kruskal–Wallis and Fisher’s exact tests, with statistical significance level set at 0.05. Data analysis was conducted and reviewed in a blinded manner. **Results:** A total of 28 studies published between 2015 and 2024 met the inclusion criteria. A total of 2082 patients were included, with 323 (41.9%) of the 770 cases testing positive for HPV and 327 (24.9%) of the 1312 controls testing positive for HPV. A total of 18 studies specifically examined HPV genotypes. Cervical swabs were employed in 19 out of 28 studies (67.9%), while vaginal swabs were used in 17 studies (60.7%). Additionally, two studies included samples collected via cervical biopsy (7.1%), four studies utilized cervicovaginal lavage (14.3%), and one study used a cervical brush for sample collection (3.6%). Regarding microbiota profiling, 26 studies (92.9%) employed 16S rRNA analysis, while one study (3.6%) utilized whole-genome sequencing (WGS), and another (3.6%) used 16s rDNA. A total of 10 studies (35.7%) examined the distribution of CSTs. Five studies (17.9%) reported on *Lactobacillus* profiles. Different levels of *Lactobacillus crispatus* and *Lactobacillus iners* were observed, along with variations between *Lactobacillus*-dominant and *Lactobacillus*-depleted communities. A total of 22 studies (78.6%) assessed α-diversity, and 17 studies (60.7%) examined β-diversity; **Conclusions:** This study emphasizes the heterogeneous features of the studies exploring the association between alterations in the CVM, HPV, and the development of ICC, suggesting the need for further research to better understand this relationship. These findings could inform new strategies for prevention and treatment.

## 1. Introduction

In recent years, the study of cervicovaginal microbiota (CVM) has gained significant attention due to the growing body of evidence highlighting its crucial role in women’s health [1]. Among women of reproductive age, it is predominantly dominated by *Lactobacillus* species, with four primary species commonly identified: *Lactobacillus crispatus*, *Lactobacillus gasseri*, *Lactobacillus iners*, and *Lactobacillus jensenii* [2]. These *Lactobacillus* species utilize carbohydrates from the host’s mucosal epithelial cells to produce lactic acid, which inhibits the adhesion, colonization, and growth of pathogenic bacteria, thereby ensuring the stability and resilience of the microbial ecosystem [3]. The importance of a *Lactobacillus*-dominated environment as a hallmark of women’s health is underscored by the classification system proposed by Ravel et al., which categorizes vaginal microbial profiles into five distinct community state types (CSTs) based on hierarchical taxonomic clustering. CSTs I, II, III, and V are characterized by the dominance of *Lactobacillus crispatus*, *Lactobacillus gasseri*, *Lactobacillus iners*, and *Lactobacillus jensenii*, respectively. However, in some women, the microbiota is not *Lactobacillus*-dominant and is instead characterized by a diverse mixture of anaerobic and microaerophilic bacteria, such as Gardnerella, Atopobium, Prevotella, and Sneathia, which corresponds to CST IV [4]. This particular community type is associated with a state of dysbiosis, which has significant implications for women’s health [5]. Microbiota disorders compromise cervicovaginal barrier function, facilitating the adhesion, invasion, and colonization of pathogenic flora [3]. This disruption also alters the metabolic profile of the vaginal environment, leading to an increased risk of inflammation [6]. Importantly, high genital inflammation has been linked to the persistence of Human Papillomavirus (HPV), a known critical factor in the progression from infection to cervical dysplasia and malignancy [7].

Laboratory techniques for profiling *Lactobacillus* and other cervicovaginal microflora have been crucial in understanding these microbial ecosystems. The most widely used method in recent studies is 16S rRNA gene sequencing, which allows for identifying and quantifying bacterial species. This method amplifies conserved regions of the bacterial ribosomal RNA gene, providing a comprehensive overview of the microbial diversity. Whole-genome sequencing (WGS) has also been used in some studies to gain deeper insights into the genetic and functional characteristics of both dominant and minor microbial populations. In addition, quantitative polymerase chain reaction (qPCR) has been employed to specifically quantify certain bacterial species, including *Lactobacillus* and key pathogens involved in dysbiosis.

In addition to the *Lactobacillus* profile, microbial diversity may play a significant role in determining women’s health [8]. The concepts of CVM α-diversity (within a single microbial community) and β-diversity (between different microbial communities) are critical in understanding the complexity of the genital ecosystem. Higher α-diversity has been associated with dysbiosis and increased susceptibility to infections, including HPV. In contrast, lower β-diversity, indicating less variation between different microbial communities, may reflect a more stable and healthier microbiota.

Therefore, differences in the CVM compared to its physiological state may underlie the pathogenic mechanisms of various genital disorders. Among these, invasive cervical cancer (ICC) is one of the most prevalent and lethal gynecological malignancies worldwide. There is increasing interest in defining the characteristics of the CVM in ICC, as evidence indicates that these may differ in affected women and potentially interact with HPV infection, acting as cofactors. Additionally, some studies suggest that ICC itself may disrupt the balance between commensal and pathogenic microbes, further complicating the relationship between the microbiota and disease progression [9]. Understanding these correlations is essential not only for early detection and prevention but also for optimizing treatment strategies. However, despite the growing interest in this field, the evidence regarding these characteristics of CVM in ICC remains limited and often conflicting. Variability in study designs, populations, and methods of microbial analysis—such as the type of sample collected, and the techniques used for microbial profiling—contributes to these inconsistencies.

Given these considerations, we conducted a systematic review focusing on the main characteristics of available studies exploring the association between CVM diversity, *Lactobacillus* profiles, and HPV in ICC.

## 2. Materials and Methods

The systematic review was registered in the INPLASY database with registration number INPLASY202530027 to ensure transparency and adherence to best methodological practices. The research strategy was decided a priori, following the Preferred Reporting Items for Systematic Reviews and Meta-Analyses (PRISMA) [10]. Since published de-identified data were used, this study was exempt from institutional review board approval.

**Figure 1:**
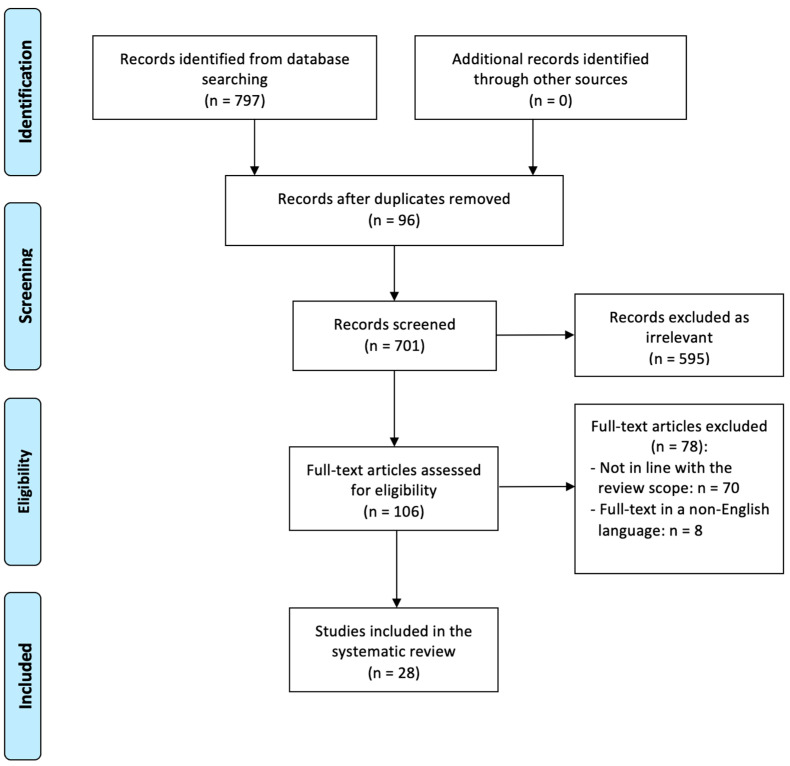
PRISMA flow diagram.

A literature search was systematically performed across the following databases: Medline, Embase, Scopus, the Cochrane Database of Systematic Reviews, and ClinicalTrials.gov., evaluating the available articles from inception to August 2024. For each database, we retrieved all articles using the following search strategy: ((microbiota[Title/Abstract] OR microbiome[Title/Abstract]) AND (cervical cancer[Title/Abstract] OR cervical carcinoma[Title/Abstract])). The PRISMA flow diagram (Figure 1) summarizes the search strategy.

All studies evaluating the CVM of patients affected by cervical cancer—in terms of CSTs, *Lactobacillus* profiles, α-diversity, and β-diversity—compared with healthy patients have been included in the final analysis.

### 2.1. Study Selection

Study selection was made independently by two authors (G.G.I. and S.C.). In case of discrepancy, a third author (C.R.) decided on inclusion or exclusion. Inclusion criteria were: (1) studies that included patients with full information about the profile of CVM and at least one group with HPV infection; (2) studies with full information about methods of profiling (3) peer-reviewed articles published originally. We excluded non-original studies, preclinical trials, animal trials, abstract-only publications, and articles in a language other than English. If possible, the authors of studies that were only published as congress abstracts were tried to be contacted via email and asked to provide their data. We assessed all included studies regarding potential conflicts of interest.

### 2.2. Statistical Analysis

All the variables were previously graphical as histograms and examined for parametric or non-parametric distribution. Continuous variables were expressed as median and interquartile range and compared using the Kruskal–Wallis test due to the non-parametric distribution. Dichotomous and Ordinal variables were expressed as absolute numbers and percentages and compared using Fisher’s exact test. No subgroup analysis was performed. The statistical significance level was set at 0.05, and all statistical investigations were performed using R software and R Studio vers. 2023.12.1 + 402.

### 2.3. Risk of Bias

Data analysis was conducted first by C.R. and then by blinding by G.V., who was unaware of the study’s objective. No missing data were present in the outcomes of interest.

## 3. Results

Figure 1 summarizes the process of literature identification and selection of the studies. The systematic bibliographic research strategy identified 797 studies, from which 96 duplicates were removed. After a review of the titles and abstracts, 108 full-text records were assessed for eligibility. Finally, 28 studies met the inclusion criteria [11,12,13,14,15,16,17,18,19,20,21,22,23,24,25,26,27,28,29,30,31,32,33,34,35,36,37,38], whose characteristics are shown in Table 1.

The studies included were published between 2015 and 2024. The studies were conducted in various countries, with the highest number of studies coming from China (14 studies, 50%). A total of 2082 patients were included in the analysis, with 323 (41.9%) of the 770 cases testing positive for HPV and 327 (24.9%) of the 1312 controls testing positive for HPV.

Eighteen studies specifically examined HPV genotypes.

The studies utilized various sample types and collection methods to analyze the CVM. Cervical swabs were the most used sample type, employed in 19 out of 28 studies (67.9%), while vaginal swabs were used in 17 studies (60.7%). Additionally, two studies included samples collected via cervical biopsy (7.1%), four studies utilized cervicovaginal lavage (14.3%), and one study used a cervical brush for sample collection (3.6%).

Regarding microbiota profiling, the majority of studies (26 out of 28, 92.9%) employed 16S rRNA analysis, while one study (3.6%) utilized WGS, and another (3.6%) used 16s rDNA. A total of 10 studies (35.7%) examined the distribution of CSTs. The studies reported various microbial profiles, with CST IV being identified in multiple samples. Five studies (17.9%) specifically reported on *Lactobacillus* profiles. Across these studies, different levels of *Lactobacillus crispatus* and *Lactobacillus iners* were observed, along with variations between *Lactobacillus*-dominant and *Lactobacillus*-depleted communities.

A total of 22 studies (78.6%) assessed α-diversity, and 17 studies (60.7%) examined β-diversity.

## 4. Discussion

### 4.1. Cervical Cancer and Microbiota

ICC is one of the most common gynecological cancers, with nearly all cases being linked to HPV [39]. However, while HPV is essential for the development of cancer, it alone is not sufficient [40], as only 0.6–3% of individuals with HPV infection progress to ICC [41]. This suggests that other contributing factors are involved in the carcinogenic process [42]. Although the exact mechanisms behind HPV persistence and the development of cervical neoplasms are not fully understood, it is clear that a range of factors influence this process, including host-related elements like immune status, smoking habits, parity, and sexual behavior; mechanical influences such as vaginal douching; and other biological factors, including sexually transmitted infections [7]. Among these, some studies have focused on the CVM, finding that individuals with a microbiota composition lacking *Lactobacillus* species or dominated by *Lactobacillus iners* (as seen in CST IV) have more than double the risk of HPV infection [43]. For example, a study involving 68 HPV-discordant monozygotic female Korean twins found that the HPV-positive twins had lower levels of *Lactobacillus* spp. and increased levels of Fusobacteria and Sneathia spp. compared to their HPV-negative twins [44]. This evidence suggests that an abnormal CVM may play a significant role in the onset of cervical neoplasms [45], similar to findings from other research that connect specific bacterial species to the development of various cancers [46]. From a pathogenic perspective, bacteria associated with dysbiosis have been linked to genital inflammation, which may encourage carcinogenesis. In fact, one study found elevated levels of proinflammatory cytokines in patients with cervical dysplasia [47]. Supporting this, research conducted among South African women examining HPV, vaginal dysbiosis, and cervical intraepithelial neoplasia grade 2 or higher (CIN2+) found that acquiring HPV alters the CVM. Moreover, the likelihood of anaerobic dysbiosis seems to rise in parallel with the development of CIN2+, indicating that the vaginal microbiome’s composition could mediate CIN2+ development through persistent HPV infection [48]. Additionally, other studies have indicated that *Lactobacillus iners* populations tend to increase in HPV-positive women and in those with cervical dysplasia [49].

### 4.2. Cervical Cancer and Community State Types

CST IV, typically dominated by anaerobic bacteria such as Gardnerella vaginalis, Atopobium vaginae, and Sneathia, seems to be frequently associated with bacterial vaginosis and heightened inflammation, which may support HPV persistence and promote oncogenic transformation [42]. In line with this, a longitudinal analysis of vaginal swabs collected over 16 weeks from 32 sexually active women found that CST IV is linked to the slowest regression of HPV, while CST II is associated with the most rapid regression rates [50]. These findings underscore the importance of understanding how specific CSTs influence HPV infection and its progression. Such insights could lead to the development of microbiota-based biomarkers, aiding in early detection and risk stratification.

### 4.3. Cervical Cancer and Lactobacillus Profiles

*Lactobacillus crispatus* is well known for its ability to produce lactic acid, which maintains vaginal pH at levels inhospitable to many pathogens, including HPV [3]. The depletion of *Lactobacillus* species in ICC cases suggests that these beneficial bacteria are crucial in preventing HPV persistence and the subsequent progression to malignancy. This finding highlights the potential for therapeutic interventions aimed at reinforcing the *Lactobacillus* population within the microbiota. For instance, targeted probiotics, prebiotics, or even microbiota transplantation could be explored as complementary strategies to enhance the effectiveness of existing preventive measures, such as HPV vaccination and regular screening. Some studies have demonstrated a significant positive effect of probiotics on the clearance of HPV and the regression of low-grade lesions [51]. Similarly, a longitudinal cohort study confirmed that the composition of genital tract flora is significantly associated with the regression of cervical intraepithelial neoplasia (CIN). Women with precancerous lesions dominated by lactic acid bacteria were more likely to experience regression within one year. Conversely, *Lactobacillus* depletion and specific anaerobic overgrowth were closely linked to the slower elimination of cervical lesions [52].

### 4.4. Cervical Cancer and Microbiota Diversity

α- and β-diversity are linked to the changes in the microbiota. Higher α-diversity in ICC cases may indicate a more heterogeneous and less stable microbial community, possibly reflecting a state of dysbiosis. In contrast, healthy controls generally show lower α-diversity, indicative of a stable, *Lactobacillus*-dominated microbiota. The distinct β-diversity patterns seen in ICC cases point to specific shifts in microbial composition associated with cancer development. These shifts might involve the overgrowth of pathogenic or opportunistic bacteria, exacerbating inflammation and creating a microenvironment favorable to HPV persistence and malignant transformation. The consistent differences in β-diversity reported across multiple studies highlight the need for further research to identify the key microbial players driving these changes. Gaining a deeper understanding of the specific microbial interactions and pathways involved could pave the way for the development of novel biomarkers for early ICC detection. Additionally, these findings suggest that the microbiota itself could be a target for therapeutic intervention. For instance, treatments aimed at modulating the microbiota to restore a more protective, *Lactobacillus*-dominant environment might help reduce the risk of HPV persistence and progression to ICC. However, studies on cervical dysplasia have yielded varied and sometimes contradictory results. For example, Mitra et al. found that the severity of CIN could be linked to an increase in vaginal microbiota diversity [52]. In a case–control study comparing the cervical microbiota of healthy individuals with that of patients diagnosed with CIN2/3 or ICC, microbial richness was significantly higher in the CIN2/3-ICC group than in the control group, accompanied by an increase in the number of operational taxonomic units (OTUs) [53]. Conversely, another study investigating the relationship between cervical microbiota and CIN2+ in women with HPV found no association between the α- and β-diversity of the vaginal microbiota and either CIN severity or oxidative DNA damage [54]. This variability in findings may be attributed to differences in the sequencing methods used across studies.

The range of sample types and collection methods used across studies underscores the challenges inherent in studying the CVM. While cervical and vaginal swabs are frequently employed due to their non-invasive nature and ease of collection, the use of cervical biopsies and cervicovaginal lavage reflects efforts to gather more detailed and representative microbiota samples. Cervical biopsies, for example, can provide a deeper understanding of the interaction between the microbiota and the cervical epithelium, which is the primary site for HPV infection and subsequent transformation. On the other hand, cervicovaginal lavage allows for the collection of a diverse array of microbial species from both the cervix and vagina, potentially offering a more comprehensive perspective on the CVM. The studies included in this review employed various sampling methods, with cervical swabs being the most frequently used (67.9%), followed by vaginal swabs (60.7%). A smaller proportion of studies used cervicovaginal lavage (14.3%) or cervical biopsies (7.1%), and these differences in sampling techniques likely contributed to the variability in microbial profiles observed, particularly regarding *Lactobacillus* abundance and CSTs. This variety in sampling techniques highlights the need for standardized protocols in microbiota research, as differences in how samples are collected can significantly alter the microbial profiles observed.

Moreover, the lack of consistency in laboratory methods, such as using 16S rRNA sequencing versus WGS, further complicates the comparability of results across studies. 16S rRNA sequencing was the predominant method employed in 92.9% of the studies reviewed, while WGS was used in only one study (3.6%). The widespread use of 16S rRNA sequencing as the primary method for microbiota profiling highlights its effectiveness and reliability in identifying bacterial communities by targeting the hypervariable regions of the 16S rRNA gene. However, this method is limited in providing detailed functional information about the microbiota. WGS, in contrast, has the potential to provide a deeper insight into the microbiota’s full genetic content, including the detection of virulence factors, antibiotic resistance genes, and metabolic pathways that may influence cancer progression. Despite its clear advantages, the relatively infrequent use of WGS suggests that practical challenges, such as higher costs, increased computational demands, and longer analysis times, may limit its widespread application. Nevertheless, WGS could provide invaluable insights into the functional dynamics of the CVM that go beyond taxonomic profiling, potentially revealing mechanisms of interaction between the microbiota and the host that contribute to cervical cancer development. To gain a more thorough understanding of the functional roles of the CVM in ICC, future research should aim to integrate 16S rRNA sequencing with WGS or other metagenomic techniques. This would enable researchers to both accurately characterize microbial communities and assess the functional potential of the microbiota in influencing disease outcomes.

### 4.5. Cervical Cancer and Human Papillomavirus

Despite HPV detection being a cornerstone of cervical screening, there remain challenges in effectively stratifying HPV-positive patients according to lesion risk. The current HPV testing is constrained by various factors, including its significant dependence on human interpretation [55]. To address these limitations, it may be advantageous to combine HPV testing with analysis of CVM characteristics if an interplay exists, which could be conducted on the same clinical specimen within a single diagnostic pipeline. This combined approach could potentially enhance the positive predictive value in managing cervical disease. Noninvasive microbiome models have already been successfully developed for the diagnosis of several cancer types, including colorectal, breast, and liver cancers [56]. In a similar vein, the composition of the CVM could be hypothesized as a valuable biomarker of ICC. Previous studies suggest that *Lactobacillus* species may serve as effective biomarkers for predicting HPV infection, while various pathogenic anaerobic and aerobic bacteria could be potential biomarkers for the prediction of cervical lesions [44]. Specifically, *Lactobacillus crispatus* has been consistently associated with a stable and healthy cervicovaginal microbiota, which may protect against HPV persistence and the development of cervical lesions. In contrast, the depletion of *Lactobacillus crispatus* and the predominance of *Lactobacillus iners* or other anaerobic species, as observed in CST IV, have been linked to dysbiosis, increased inflammation, and higher risks of HPV infection and progression to CIN or ICC. Given these associations, *Lactobacillus* species, particularly *Lactobacillus crispatus*, could be explored as biomarkers not only for the presence of a healthy CVM but also for stratifying the risk of cancer progression. Further research is needed to validate the use of *Lactobacillus* as a clinical biomarker, considering its potential application in noninvasive screening tools for early detection of HPV-related lesions. The ability to monitor shifts in *Lactobacillus* abundance and diversity could aid in identifying individuals at higher risk for developing ICC, enabling earlier intervention and potentially improving patient outcomes.

### 4.6. Strengths and Limitations

This study has several strengths, including a comprehensive literature review conducted in strict accordance with PRISMA guidelines. The inclusion of studies spanning nearly a decade, from 2015 to 2024, offers a thorough overview of the evolving research landscape regarding the role of the CVM in ICC. Additionally, the large sample size of patients from diverse populations and settings further enhances the comprehensiveness of this review.

However, several limitations must be acknowledged. This study was limited to summarizing the main characteristics of the existing literature. The variability in sample types and collection methods across the included studies, coupled with the relatively small number of studies focusing on specific elements, like CSTs and *Lactobacillus* profiles, restricts the ability to perform robust statistical evaluations of associations. The inherent complexity of the microbiota-cancer relationship further complicates the establishment of definitive connections. Additionally, environmental factors, such as treatment interventions and disease progression, may introduce confounding variables, necessitating a cautious interpretation of the findings. The diversity in microbial compositions and pathogen-specific responses adds further complexity to the analysis, highlighting the critical need for standardized methodologies and larger-scale studies to elucidate these relationships definitively.

## 5. Conclusions

In conclusion, this study emphasizes the heterogeneous features of the studies exploring the association between alterations in the CVM, HPV, and the development of ICC, highlighting the need for further research to better understand this relationship. Future studies should aim to standardize methodologies and incorporate longitudinal assessments to gain a deeper understanding of the intricate connections between the CVM and ICC, ultimately enhancing prevention and treatment strategies.

## Figures and Tables

**Table 1:** Characteristics of the studies included in the systematic review.

Author, Year	Country	Cases (n)	Controls (n)	HPV Genotypes	Cases+ (n, %)	Controls+ (n, %)	Sample Type	Microbial Analysis	CSTs Cases (n, %)	CSTs Controls (n, %)	*Lactobacillus* Profiles Cases (n, %)	*Lactobacillus* Profiles Controls (n, %)	α-Diversity (Index)	β-Diversity (Index)
Audirac-Chalifour, 2016 [11]	Mexico	8 ICC+	10 NILM-, 10 NILM+	NR	8 (100)	10 (50)	cervical (swab, biopsy)	V3-V4 16S rRNA	IV: 2 (25), VI: 1 (12.5), VII: 2 (25), VIII: 3 (37.5)	NILM-: I: 4 (57), II: 1 (14), V: 1 (14), VI: 1 (14); NILM+: I: 2 (20), II: 4 (40), III: 3 (30), V: 1 (10)	NR	NR	↔ cases 3.08 ± 1.28 vs. NILM- 2.00 ± 0.63: *p* = 0.498, ↔ cases 3.08 ± 1.28 vs. NILM+ 2.49 ± 0.70: *p* = 1 (Shannon index), ↑ cases 4.14 ± 1.49 vs. NILM- 1.55 ± 0.99: *p* = 0.036, ↔ cases 4.14 ± 1.49) vs. NILM+ 2.49 ± 1.61): *p* = 0.318 (PD whole tree)	*p* < 0.00001 (cases vs. NILM-) (weighted Unifrac)
Chen, 2020 [12]	China	9 ICC+	68 NILM-, 78 NILM+	NR	9 (100)	78 (53.4)	vaginal (swab)	V3-V4 16S rRNA	III: 1 (11.1), IV: 8 (88.9)	NILM-: I: 14 (20.6), II: 2 (2.9), III: 32 (47.1), IV: 20 (29.4); NILM+: I: 14 (17.9), II (2.6), III: 28 (35.9), IV: 32 (41.0), V: 2 (2.6)	NR	NR	↑ cases: 367.76 ± 208.63 vs. NILM-: 84.02 ± 73.88 (q ≤ 0.001) vs. NILM+: 272.26 ± 191.62 (Chao index); cases: 2.47 ± 0.98 vs. NILM-: 0.94 ± 0.95 (q ≤ 0.001) vs. NILM+: 1.49 ± 1.01 (q < 0.05) (Shannon index)	cases vs. NILM-: R = 0.284, *p* = 0.001; cases vs. NILM+: R = −0.0359, *p* = 0.656 (Unweighted Unifrac)
Fan, 2021 [13]	China	65 ICC	54 NILM	16, 18, 31, 33, 52, 58, 35, 39, 45, 51, 56, 59, 68	63 (96.9)	47 (87)	vaginal (swab)	V3-V4 16S rRNA	NR	NR	NR	NR	↑ *p* < 0.0001 (Chao1, Shannon, Simpson, OTUs)	*p* < 0.05
Han, 2024 [14]	China	84 ICC	180 NILM	NR	NR	NR	vaginal (swab)	V3-V4 16S rRNA	NR	NR	NR	NR	↔ *p* > 0.05 (Chao, Shannon, Simpson)	*p* = 0.001 (Bray–Curtis)
Ivanov, 2023 [15]	Russia	17 ICC	77 NILM	16, 18, 31, 33, 35, 39, 45, 51, 52, 56, 58, 59, 26, 53, 66, 68, 73, 82	16 (94.1)	19 (24.7)	cervical (swab)	V3-V4 16S rRNA	NR	NR	NR	NR	*p* ≤ 0.001 (Shannon, OTUs), ↑ *p* = 0.000344 (Faith’s)	NR
Kang, 2021 [16]	Korea	8 ICC	7 NILM	16, 18, 26, 31, 33, 35, 39, 45, 51, 52, 56, 58, 59, 66, 68, 69, 73, 82, 6, 11, 40, 42, 44, 53, 54, 70	8 (100)	0 (0)	vaginal (swab)	V3 16S rRNA	NR	NR	NR	NR	↑ *p* = 0.0012 (Richness index), ↔ *p* > 0.05 (Shannon index), ↔ *p* > 0.05 (Simpson index)	*p* = 0.001 (Bray–Curtis)
Kwom, 2018 [17]	Korea	12 ICC	18 NILM	NR	NR	NR	cervical (swab)	Whole-genome sequencing	NR	NR	NR	NR	*p* = 0.1218 (Shannon), ↔ *p* = 0.0863 (Simpson)	*p* = 0.087 (Bray–Curtis), *p* = 0.094 (Jaccard)
Łaniewski, 2018 [18]	USA	10 ICC	51 NILM	16, 18, 31, 33, 35, 39, 45, 51, 52, 56, 58, 59, 68	9 (90)	31 (60.8)	cervical (swab, lavage)	V4 16S rRNA	NR	NR	LDo: 2 (20), LDe: 8 (80)	NILM-: LDo: (60), LDe: (40); NILM+: LDo: (68), LDe: (32)	NR	NR
Li C, 2022 [19]	China	6 ICC+	25 NILM-	NR	6 (100)	0 (0)	cervical (swab)	V3-V4 16S rRNA	I: (40.9), II: (4.6), III: (31.8), IV: (18.2), V: (4.5)	II: (50), IV: (50)	NR	NR	NR	*p* = 0.044 (unweighted Unifrac)
Li X, 2023 [20]	China	79 ICC	79 NILM-, 80 NILM+	16, 18, 31, 33, 35, 39, 45, 51, 52, 56, 58, 59, 66, 68, and 13 LR	NR	NR	vaginal (swab)	V3-V4 16S rRNA	NR	NR	NR	NR	↑ *p* < 0.01 (Chao, Shannon, Simpson, OTUs, Pielou)	NR
Li Y, 2023 [21]	China	26 ICC	53 NILM	16, 18, 31, 33, 35, 39, 45, 51, 52, 56, 58, 68, 26, 53, 66, 73, 82, 6, 11, 81	NR	NR	vaginal (swab)	V4 16S rRNA	III: 7 (26.9), IV: 19 (73.1)	I: 15 (28.3), III: 18 (34), IV: 18 (34), V: 1 (1.9)	LDo: 7 (26.9), LDe: 19 (73.1)	LDo: 33 (62.3), LDe: 19 (35.8)	↑ *p* < 0.05 (Chao), ↔ *p* = 0.065 (Shannon)	*p* = 0.002 (Bray–Curtis)
Liu, 2022 [22]	China	41 ICC+	34 NILM+	16, 18, 26, 31, 33, 35, 39, 45, 51, 52, 53, 56, 58, 59, 66, 68, and 82	41 (100)	34 (100)	cervical (swab)	16S rRNA	III: 4 (9.8), IV: 37 (90.2)	I: 9 (26.5), III: 14 (41.2), IV: 11 (32.3)	LDo: 4 (9.8), LDe: 37 (90.2)	LDo: 23 (67.7); LDe: 11 (32.3)	↑ *p* < 0.05 (Chao index), ↑ *p* < 0.001 (Shannon index)	R = 0.109, *p* = 0.001 (Bray–Curtis)
Ma, 2023 [23]	China	27 ICC	30 NILM-, 22 NILM+	16, 18, 31, 33, 35, 39, 45, 51, 52, 56, 58, 59, 68, 26, 53, 66, 73, 82, 6, 11, and 81	21 (77.8)	22 (42.3)	vaginal (swab)	V4 16S rRNA	III: 7 (25.9), IV: 20 (74.1)	HPV-: I: 9 (30), III: 10 (33.3), IV: 10 (33.3), V: 1 (3.3); HPV+: 6 (27.3), III: 8 (36.4), IV: 12 (26.7), V: 2 (4.4)	LDo: 7 (25.9), LDe: 20 (74.1)	NILM-: LDo: 19 (63.3), LDe: 11 (36.7); NILM+: LDo: 14 (63.6), 8 (36.4)	↔ NILM- vs. NILM+ (*p* > 0.05) vs. ↑ cases (*p* < 0.01) (Shannon index); ↔ NILM- vs. NILM+ (*p* > 0.05) vs. ↑ cases (*p* < 0.05) (Simpson index); ↔ NILM- vs. NILM+ (*p* > 0.05) vs. ↑ cases (*p* < 0.001) (Sobs)	NR
Mitra, 2015 [24]	England	20 ICC	5 NILM	16, 18, 12, 31, 33, 35, 39, 45, 51, 52, 56, 58, 59, 66, 68	NR	NR	vaginal (swab)	V1-V2 16S rRNA	I: 1 (20), II: 1 (20), IV: 2 (40), V: 1 (20)	I: 10 (50), III 8 (40), IV: 2 (10)	NR	NR	NR	NR
Musa, 2023 [25]	Nigeria	30 ICC	19 NILM	16, 18, 26, 31, 33, 35, 39, 45, 51, 52, 53, 56, 58, 59, 66, 68, 69, 73, 82, 6, 11, 40, 42, 43, 44, 54, 61, 70	27 (90)	8 (42.1)	cervico- vaginal (lavage)	V3-V4 16S rRNA	I: 2 (0.7), III: 3 (10), IV: 25 (83.3)	I: 2 (10.5), III: 8 (42.1), IV: 9 (47.4)	NR	NR	NR	NR
Ou, 2024 [26]	China	25 ICC	10 NILM	NR	9 (90)	22 (88)	vaginal (swab), cervico- vaginal (lavage)	V3-V4 or V4-V5 16S rRNA	NR	NR	NR	NR	NR	*p* < 0.001 (Bray–Curtis)
Sekaran, 2023 [27]	India	65 ICC	54 NILM	NR	NR	NR	vaginal (swab), cervico- vaginal (lavage)	16S rRNA	NR	NR	NR	NR	NR	*p* = 0.001 (Bray–Curtis)
Stoian, 2023 [28]	Romania	9 ICC+	20 NILM-, 9 NILM+	16, 18, 31, 33, 35, 39, 45, 51, 52, 56, 58, 59, 68	9 (100)	9 (31)	cervical (swab)	V3-V4 16S rRNA	NR	NR	NR	NR	↑ *p* = 0.0019 (Shannon)	NR
Teka, 2023 [29]	Ethiopia	60 ICC	35 NILM	16, 18, 26, 31, 33, 35, 39, 45, 51, 52, 53, 56, 58, 59, 66, 68, 69, 73, 82, 6, 11, 40, 42, 43, 44, 54, 61, 70	NR	NR	cervical (swab, brush)	V4 16S rRNA	NR	NR	NR	NR	↑ *p* = 0.00000054 (Shannon), *p* = 0.000005 (Simpson)	*p* = 0.001 (weighted UniFrac)
Wang, 2022 [30]	China	26 ICC	40 NILM	16, 18, 11, 31, 33, 35, 39, 45, 51, 52, 56, 58, 59, 66, 68,	18 (69.2)	1 (2.5)	vaginal (swab)	V3-V4 16S rRNA	NR	NR	NR	NR	↑ *p* < 0.001 (Shannon), ↓ *p* < 0.001 (Simpson)	R = 0.464, *p* = 0.001 (Bray–Curtis)
Wei, 2022 [31]	China	11 ICC	10 NILM-, 13 NILM+	12, 16, 18, 31, 33, 35, 39, 45, 51, 52, 56, 58, 59, 66, 68	11 (100)	13 (56.5)	cervical (biopsy)	V3-V4 16S rRNA	I: 1 (9.1), II: 7 (63.6), III: 3 (27.3)	NILM-: I: 6 (60), II: 4 (40); NILM+: I: 5 (38.5), II: 5 (38.5), III: 3 (23.1)	NR	NR	NILM- vs. ↑ NILM+: *p* = 0.03971 vs. ↑ cases: *p* = 0.004151 (Shannon); NILM- vs. ↑ NILM+: *p* = 0.01851 vs. ↑ cases: *p* = 0.000894 (Simpson)	NR
Wu, 2021 [32]	China	13 ICC	28 NILM-, 12 NILM+	16, 18, 31, 33, 35, 39, 45, 51, 52, 56, 58, 59, 66, 68, 53, 6, 11, 42, 43, 44, CP8304(81)	10 (76.9)	12 (30)	cervical (swab)	V4 16S rRNA	II: 11 (85), III: 2 (15)	NILM-: NR; NILM+: II: 10 (83), 2 (17)	NR	NR	↑ *p* < 0.05 (Shannon, Simpson)	*p* < 0.01 (weighted Unifrac)
Xie, 2020 [33]	China	18 ICC+	25 NILM-	16, 18, 26, 31, 33, 35, 39, 45, 51, 52, 53, 56, 58, 59, 66, 68, 73, 82, 6, 11, 40, 42, 43, 44, 54, 61, 81, 83	18 (100)	0 (0)	vaginal (swab)	V4 16S rRNA	NR	NR	LDo: 3 (18.4), LDe: 15 (81.6)	LDo: 9 (35.6), LDe: 16 (64.4)	↔ *p* = 0.2609 (Shannon), *p* = 0.2245 (Simpson)	NR
Xu, 2022 [34]	China	10 ICC	10 NILM	16, 18, 31, 33, 35, 39, 42, 43, 44, 45, 51, 52, 56, 58, 59, 68	NR	NR	cervico- vaginal (swab)	V3-V4 16S rRNA	NR	NR	NR	NR	↑ *p* = 0.04 (Shannon), *p* = 0.02 (Simpson)	F = 1.8557, R2 = 0.1407, *p* = 0.008 (Bray–Curtis)
Zeber-Lubecka, 2022 [35]	Poland	16 ICC	30 NILM-	NR	NR	0 (0)	cervical (swab)	V2-V3-V4-V6-V7-V8-V9 16S rRNA	NR	NR	NR	NR	premenopause: ↔ *p* = 0.055 (Chao), ↑ *p* = 0.0025 (Shannon); postmenopause: ↔ *p* = 0.7 (Chao), ↑ *p* = 0.026 (Shannon)	NR
Zeng, 2023 [36]	China	15 ICC	15 NILM	NR	NR	NR	vaginal (swab)	V3-V4 16S rRNA	NR	NR	NR	NR	↑ *p* = 0.0023 (Chao1), *p* = 0.0023 (Shannon), *p* = 0.0043 (Simpson), *p* = 0.0012 (OTUs), *p* = 0.0010 (PD whole tree), *p* = 0.0007 (goods coverage)	NR
Zhai, 2021 [37]	China	38 ICC	29 NILM-, 29 NILM+	NR	NR	NR	cervical (swab)	V3-V4 16S rRNA	NR	NR	NR	NR	↔ *p* > 0.05(Chao1, Shannon, Simpson, PD whole tree, ACE), ↓ cases vs. NILM-: *p* ≤ 0.05 (OTUs)	*p* ≤ 0.05 (weighted UniFrac)
Zhang, 2024 [38]	China	22 ICC+	22 NILM-, 21 NILM +	16, 18, 33, 51, 52, 53, 58	22 (100)	21 (48.8)	vaginal (swab)	16s rDNA	NR	NR	NR	NR	NILM- vs. ↑ NILM+: 0.013 vs. cases: ↑ 0.00055 (Chao1), NILM- vs. ↑ NILM+: *p*=0.005 vs. ↑ cases: 6.7 × 10^−7^ (Shannon), NILM- vs. ↑ NILM+: 0.0039 vs. ↑ cases: 1.3 × 10^−6^ (Simpson)	R2 = 0.189, *p* = 0.001 (unweighted UniFrac), R2 = 0.05, *p* = 0.017 (weighted UniFrac)

CST, community state type; ICC, invasive cervical cancer; LDe, Lactobacilli-depleted; LDo, Lactobacilli-dominant; n, number; NILM, negative for intraepithelial lesion or malignancy; NR, not reported; PD, phylogenetic diversity. “-” means HPV-negative; “+” means HPV-positive; “↑” means that the index (or indices) is higher in cases than in controls, “↓” means that the index (or indices) is lower in cases than in controls, “↔” means no significant difference between the groups.

## Data Availability

The data that support the findings of this study are available on request from the corresponding author.
